# Characterization of Hair Metabolome in 5xFAD Mice and Patients with Alzheimer’s Disease Using Mass Spectrometry-Based Metabolomics

**DOI:** 10.1021/acschemneuro.3c00587

**Published:** 2024-01-25

**Authors:** Chih-Wei Chang, Jen-Yi Hsu, Yu-Tai Lo, Yu-Hsuan Liu, Onanong Mee-inta, Hsueh-Te Lee, Yu-Min Kuo, Pao-Chi Liao

**Affiliations:** † Department of Environmental and Occupational Health, College of Medicine, National Cheng Kung University, Tainan 704, Taiwan; ‡ Department of Geriatrics and Gerontology, National Cheng Kung University Hospital, College of Medicine, National Cheng Kung University, Tainan 704, Taiwan; § Department of Public Health, College of Medicine, National Cheng Kung University, Tainan 704, Taiwan; ∥ Institute of Basic Medical Sciences, College of Medicine, National Cheng Kung University, Tainan 701, Taiwan; ⊥ Department of Cell Biology and Anatomy, College of Medicine, National Cheng Kung University, Tainan 701, Taiwan; # Institute of Anatomy and Cell Biology, School of Medicine, National Yang Ming Chiao Tung University, Taipei 112, Taiwan; ∇ Department of Food Safety/Hygiene and Risk Management, College of Medicine, National Cheng Kung University, Tainan 701, Taiwan

**Keywords:** untargeted and targeted metabolomics, hair, aachidonic acid metabolism, sphingolipid metabolism, alanine metabolism

## Abstract

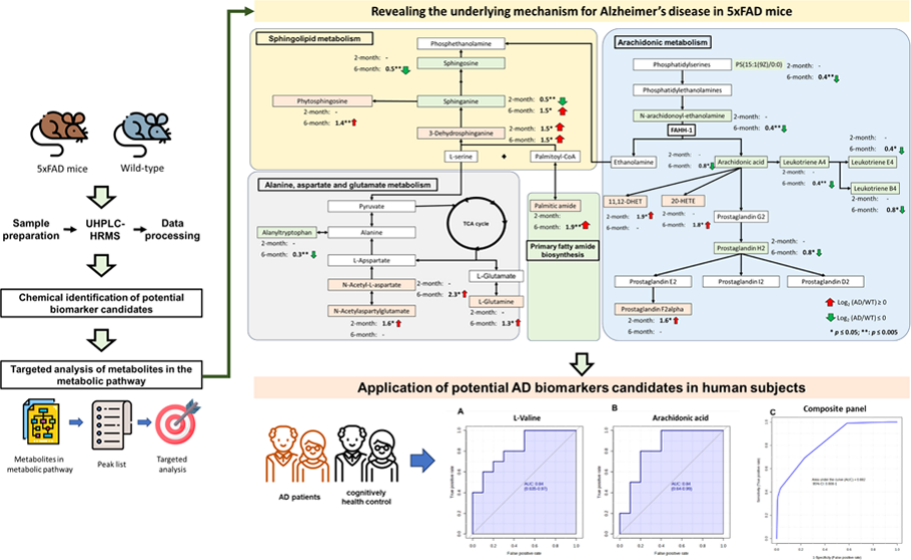

Hair emerged as a biospecimen for long-term investigation of endogenous metabolic perturbations, reflecting the chemical composition circulating in the blood over the past months. Despite its potential, the use of human hair for metabolomics in Alzheimer’s disease (AD) research remains limited. Here, we performed both untargeted and targeted metabolomic approaches to profile the key metabolic pathways in the hair of 5xFAD mice, a widely used AD mouse model. Furthermore, we applied the discovered metabolites to human subjects. Hair samples were collected from 6-month-old 5xFAD mice, a stage marked by widespread accumulation of amyloid plaques in the brain, followed by sample preparation and high-resolution mass spectrometry analysis. Forty-five discriminatory metabolites were discovered in the hair of 6-month-old 5xFAD mice compared to wild-type control mice. Enrichment analysis revealed three key metabolic pathways: arachidonic acid metabolism, sphingolipid metabolism, and alanine, aspartate, and glutamate metabolism. Among these pathways, six metabolites demonstrated significant differences in the hair of 2-month-old 5xFAD mice, a stage prior to the onset of amyloid plaque deposition. These findings suggest their potential involvement in the early stages of AD pathogenesis. When evaluating 45 discriminatory metabolites for distinguishing patients with AD from nondemented controls, a combination of l-valine and arachidonic acid significantly differentiated these two groups, achieving a 0.88 area under the curve. Taken together, these findings highlight the potential of hair metabolomics in identifying disease-specific metabolic alterations and developing biomarkers for improving disease detection and monitoring.

## Introduction

Alzheimer’s disease (AD) is a common progressive neurodegenerative disorder characterized by cognitive impairments, memory loss, and visuospatial dysfunctions. It accounts for up to 60% of dementia cases.^1^ The accumulation of extracellular amyloid plaques, formed by aggregation and deposition of amyloid β (Aβ) peptides, is a key mechanism in the onset of AD.^2^ Aβ peptides are formed through the sequential cleavage of the amyloid precursor protein by β- and γ-secretases.^3^ Genes associated with familial AD include amyloid precursor protein, presenilin 1, and presenilin 2, which play a role in amyloid plaque formation.^4^ The 5xFAD mice model, harboring three human amyloid precursor protein mutations (Swedish, Florida, and London) and two presenilin 1 mutations (M146L and L286 V), is commonly used to study the effects of Aβ accumulation.^5^ These mutations enhance the production of Aβ peptides, particularly the more aggregative and pathogenic Aβ42 variant. The precise nature of the metabolic changes influenced by the deposition of amyloid plaques remains unclear.

Metabolomics is the comprehensive study of metabolites, small molecule substrates, intermediates, and products of metabolism, within biological systems, such as cells, tissues, and organs.^6^ This field utilizes targeted and untargeted approaches to identify and quantify these molecules, thereby providing insights into the biochemical processes and pathways underpinning cellular and organismal function. Untargeted metabolomics involves the global profiling of a wide range of metabolites in biological samples without predefining specific metabolites of interest.^7^ In contrast, targeted metabolomics analyzes specific metabolites involved in certain metabolic pathways or disease processes.^8^ Integrating these two approaches allows for the identification of metabolic pathways relevant to understanding the underlying mechanism of AD and the development of novel therapeutic targets. Blood and cerebrospinal fluid are commonly employed in metabolomics research for biomarker discovery for AD.

Hair has emerged as a promising biospecimen for investigating the metabolic perturbations of body burden over extended periods.^9−14^ The hair matrix provides a retrospective measurement of the metabolome associated with months to even years.^15−17^ Furthermore, collecting hair samples is less invasive than collecting cerebrospinal fluid, making it more feasible and practical for routine sampling in clinical practices. The noninvasive nature of hair sample collection reduces patient discomfort and allows for easier and more frequent sampling. Indeed, hair has been recently applied in discovering disease biomarkers in clinical research.^9,18^ Additionally, the distribution of chemical compound concentrations along the hair shaft reflects changes in the corresponding composition during the measurement period.^10,19,20^

In this study, we aimed to perform a high-resolution mass spectrometry (HRMS)-based untargeted and targeted metabolomic approach on hair samples from 6-month-old 5xFAD mice, characterized by accumulation of amyloid plaques in the brain. The goal was to profile the key metabolic pathways related to AD present in the hair sample, thereby shedding light on the underlying mechanisms of AD. We extended our investigation to explore the potential participation of these metabolic pathways in the early stage of AD pathogenesis. Furthermore, the performance of the identified discriminatory metabolites was evaluated in terms of their ability to distinguish between patients with AD and nondemented control subjects to determine their potential as biomarkers for AD.

## Results

### Study Design

**Figure 1:**
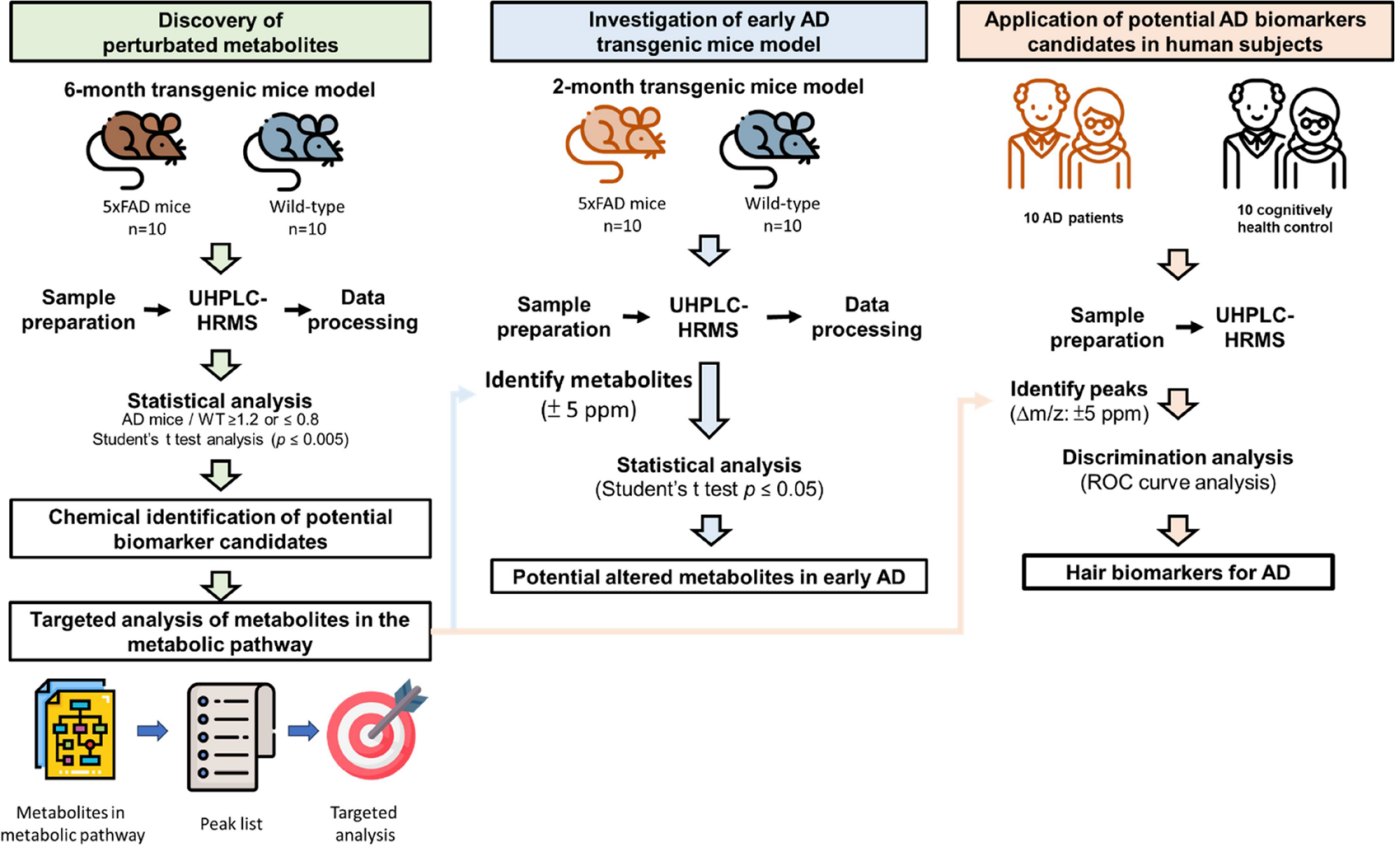
Overview of the strategies for discovering hair biomarkers for Alzheimer’s disease using the 5xAD mice model. The discriminatory metabolites were identified using untargeted and targeted metabolomics approaches in 6-month-old 5xFAD mice. The key metabolic pathways for AD were proposed and identified by enrichment and pathway analysis. The key metabolic pathways related to the early stages of AD were explored using 2-month-old 5xFAD mice. The discriminatory metabolites identified in the transgenic mice model were used to evaluate their potential as diagnostic biomarkers for AD.

The study design for the characterization of the hair metabolome alternations in hair samples of 5xFAD mice is depicted in Figure 1. The 20 hair samples collected from 6-month-old 5xFAD mice (*N* = 10) and wild-type (WT) mice (*N* = 10), along with a blank solvent sample, were subjected to the sample preparation procedure developed previously.^21^ To remove chemicals deposited on the hair surface, acetone followed by deionized water was employed to wash out these chemicals.^22^ A 50:50 phosphate-buffered saline: methanol solvent mixture at 55 °C for 4 h was used to extract metabolite in hair since it provided a wider coverage of metabolites based on previous study.^21^ Subsequently, the hair extracts, quality control (QC) prepared by pooling aliquots of hair extracts, and blank samples were analyzed using ultrahigh-performance liquid chromatography–HRMS (UHPLC–HRMS) to investigate the alternations in the hair metabolome. The QC sample was used to monitor the variation during the instrumental analysis, while the blank sample was employed to assess the noise level for background subtraction during the data processing procedure.

The HRMS data set was subsequently subjected to perform the statistical analysis. The data normality was determined by performing a Shapiro-Wilk test, and either a *t* test or Mann–Whitney *U* test was performed to discover the discriminatory metabolite candidates based on the normality results. The cutoff values of *p* ≤ 0.005 and fold change in peak area ≥1.2 or ≤0.8 were implemented. The discriminatory metabolite candidates were subjected to extra fragmentation analysis to obtain their corresponding MS/MS spectra for chemical identification.

To implement the targeted metabolomics approach, a comprehensive list of chemical compounds including all metabolites in the relevant pathways of the discriminatory metabolites was developed. The metabolites discovered by both untargeted or targeted metabolomics approaches were then subjected to pathway analysis by MetaboAnalyst 5.0 by Kyoto Encyclopedia of Genes and Genomes (KEGG) annotation, and the key metabolic pathways for AD were proposed and identified. The identified metabolites in the key metabolic pathways were further used to investigate the patterns of alternations in the early stages of AD using the 2-month-old 5xFAD and 2-month-old WT mice. The metabolic pathways associated with the early stage of AD have finally been explored.

The discriminatory metabolites identified in the transgenic mice model were used to evaluate their potential as diagnostic biomarkers for AD. Professional neurologists diagnosed patients with AD, and all subjects underwent a Montreal cognitive assessment (MoCA). By evaluating the levels of these identified metabolites in AD patients and controls, we aimed to determine their diagnostic utility and potential as biomarkers for AD. This analysis provides valuable insights into the applicability of these metabolites in clinical practice

### Untargeted Metabolomics Reveals Perturbed Metabolites in 5xFAD Mice

The hair samples of the 20 mice were subjected to the sample preparation procedure^21^ and subsequently analyzed by UHPLC-HRMS operated in positive and negative ion modes. A total of 26,161 and 19,187 aligned features from positive and negative ion modes for 20 mouse hair samples and five QC samples were processed by MS-DIAL 4.70 from the corresponding raw data to construct a peak list for further analysis, respectively (Tables S1 and S2). Twenty mice hair samples were collected from 10 6-month 5xFAD transgenic mice and 10 WT mice. Since memory dysfunctions, amyloid plaques, intraneuronal Aβ aggregates, and Aβ oligomers were demonstrated in 6-month-old 5xFAD mice, 6-month-old 5xFAD mice were selected to discover the perturbed metabolic pathway associated with AD pathogenesis. A QC sample constructed by pooling an aliquot of all mouse hair extracts was prepared to evaluate the reproducibility of the analytical method.

The principal components analysis (PCA) score plots shown in Figure S1 were employed to explain the variance within the HRMS data set by a smaller number of principal components (PCs), which are mutually uncorrelated.^23^ In the HRMS data set obtained from positive ion mode, PC1 and PC2 explained 29.3 and 25.1% of the total variance, respectively (Figure S1A). Furthermore, in the aligned features from negative ion mode, PC1 and PC2 accounted for 44.8 and 13.5% of the total variance, respectively (Figure S1B). Comparable patterns of samples are tightly clustered in the score plots. The two plots of the HRMS data set obtained from positive and negative ion modes revealed clear and tight clusters of 5 QC samples, suggesting minimal technical errors occurred in this research. However, the PCA score plots did not clearly distinguish between AD transgenic mice and WT mice.

**Figure 2:**
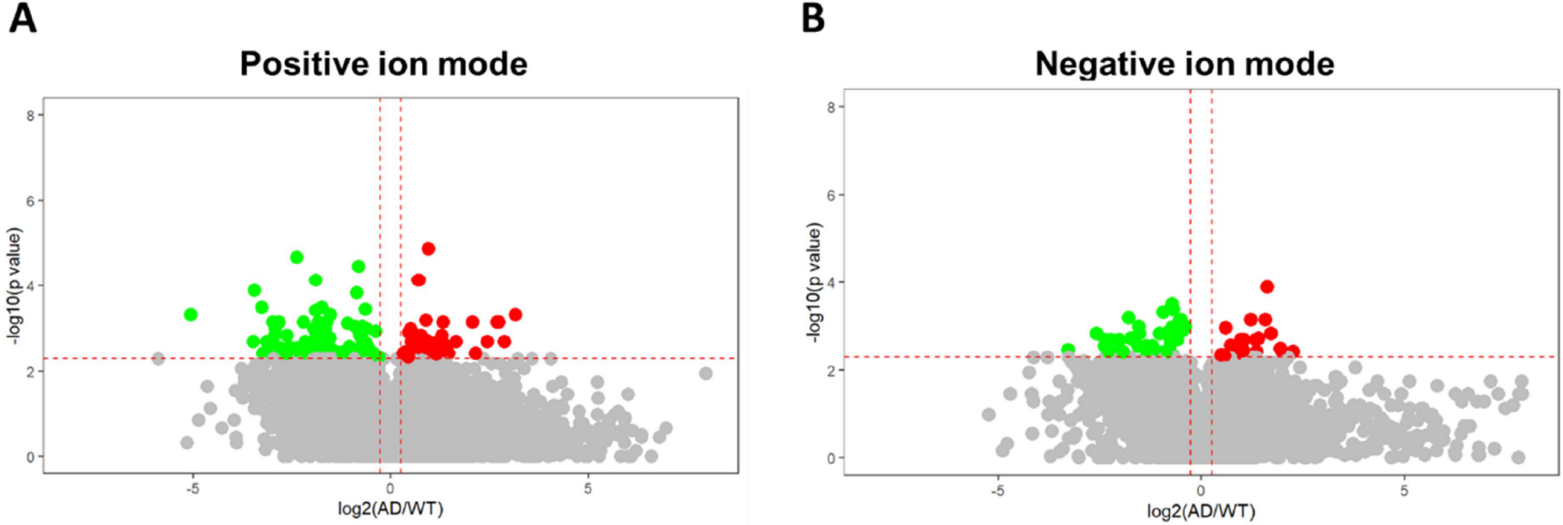
Volcano plot analysis for discovering differential metabolites in the 6-month-old 5xFAD mice. The log2-fold change (AD/WT) is plotted versus the −log10 *p*. (A) Total of 193 discriminatory features (fold change ≥1.2 or ≤0.8; *p* ≤ 0.005) were discovered between AD transgenic mice and WT mice using UHPLC-HRMS analysis operated with both positive ion modes. (B) Total of 85 discriminatory features (fold change ≥1.2 or ≤0.8; *p* ≤ 0.005) were discovered between 6-month AD transgenic mice and WT mice using UHPLC-HRMS.

To appropriately characterize the differences between 5xFAD and WT mice, the normality of each feature in the HRMS data set for these two groups was assessed using the Shapiro–Wilk test. If the *p*-value calculated by the Shapiro–Wilk test exceeded 0.05 for both groups, indicating normal distribution, Student’s *t* test was employed. On the contrary, when the *p*-value for the 5xFAD transgenic or WT mice was below 0.05, suggesting a non-normal distribution, the Mann–Whitney *U* test was employed.^24^ The normality test results for these two groups are presented in Tables S1 and S2. A volcano plot was generated with the fold change on the *x*-axis, and the *p*-value was calculated by either Student’s *t* test or Mann–Whitney *U* test on the *y*-axis to compare the two groups. Two plots revealed statistically significant differences in signal abundance between the AD transgenic and WT mice using HRMS positive and negative ion modes, based on specific criteria: fold change ≥1.2 or ≤0.8, and *p* ≤ 0.005, as shown in Figure 2A,B, respectively. A total of 278 discriminatory features were discovered, of which 84 were found in elevated levels and 194 were found in decreased levels in AD transgenic mice compared to WT mice. Subsequently, these 278 discriminatory features were subjected to fragmentation analysis for chemical identification. The corresponding experimental MS/MS spectra were extracted and further matched against a mass spectral database, including MoNA and LipidBlast or an *in silico* predicted MS/MS spectrum calculated by MS-FINDER based on chemicals deposited from databases such as HMDB, LipidMaps, and PubChem. Among these, 27 were successfully annotated to the known metabolites with a score of >70 (Table S3). The chemical structures of 2 perturbated metabolites were identified based on mass spectral database matching, and a total of 25 chemicals were identified through *in silico* predicted MS/MS spectra by MS-FINDER.

### Investigation of the Key Perturbed Metabolic Pathway in a 6-Month 5xFAD Mice Model by the Targeted Metabolomics Approach

Of the 27 chemical compounds, 10 metabolites were annotated in the KEGG database, of which 8 were identified to 6 relevant metabolic pathways using pathway analysis by MetaboAnalyst (Table S4). The most significantly enriched pathways were “sphingolipid metabolism” (*p = 0.002*) and “valine, leucine and isoleucine biosynthesis” (*p* = 0.025). The expression of the remaining five metabolic pathways was represented by chance, because they did not show significant differences compared to the random hits. The enrichment ratio values computed by observed hits/expected hits were 38.46 and 29.24 for “valine, leucine and isoleucine biosynthesis” and “sphingolipid metabolism”, respectively. While the KEGG database includes and covers a portion of metabolic pathways and metabolites, the enrichment analysis did not highlight the specific metabolic pathways related to other identified discriminatory metabolites, such as bilirubin glucuronide, N-icosanoyl ethanolamine, and alanyl-tryptophan. A literature search was conducted to identify these pathways to complement the metabolites related to AD that were not included in the KEGG database. A total of 241 chemicals are listed in Table S5 for the HRMS-based targeted metabolomics approach.

The HRMS-based targeted metabolomics approach was employed to demonstrate whether the metabolites in the two perturbed metabolic pathways participated in the pathogenesis of 6-month 5xFAD transgenic mice. A total of 18 metabolites were discovered via the targeted metabolomics approach. Among these, six discriminatory metabolites, namely sphinganine, 3-dehydrosphinganine, l-valine, l-leucine, d-erythro-3-methylmalate, and 2-methyl maleate, were identified based on the criteria: *p* ≤ 0.05 between 6-month 5xFAD transgenic mics and WT mice, as shown in Table 1. The metabolites sphinganine and 3-dehydrosphinganine, associated with the sphingolipid metabolism pathway, along with l-valine, l-leucine, d-erythro-3-methylmalate, and 2-methylmaleate, which are part of the biosynthesis pathways of “valine, leucine, and isoleucine”, were identified. Additionally, a total of 12 metabolites involved in “arachidonic acid metabolism”, “alanine, aspartate and glutamate metabolism”, and “tryptophan metabolism” that showed statistically significant differences between the 6-month-old 5xFAD and WT mice were discovered by the targeted metabolomics approach. Among these, two were involved in “alanine, aspartate, and glutamate metabolism”, six compounds were associated with “tryptophan metabolism”, and four were linked to “arachidonic acid metabolism”.

**Table 1:** Discriminatory Metabolites Discovered by the Targeted Metabolomics Approach in 6-Month 5xFAD Transgenic Mice

metabolite names	PubChem CID	chemical formula	metabolic pathway	retention time (min)	measured *m*/*z*	mass accuracy (ppm)	fold change	*p* value
sphinganine	91486	C_18_H_39_NO_2_	sphingolipid metabolism	9.0	302.3050	1.4	0.5	0.011
3-dehydrosphinganine	439853	C_18_H_37_NO_2_	sphingolipid metabolism	0.6	300.2892	1.7	1.5	0.028
l-valine	6287	C_5_H_11_NO_2_	valine, leucine and isoleucine biosynthesis	2.2	116.0703	2.4	0.5	0.005
d-erythro-3-Methylmalate	440892	C_5_H_8_O_5_	valine, leucine and isoleucine biosynthesis	0.8	147.0286	1.5	1.2	0.011
2-methylmaleate	643798	C_5_H_6_O_4_	valine, leucine and isoleucine biosynthesis	1.1	129.0179	2.0	1.2	0.009
l-leucine	6106	C_6_H_13_NO_2_	valine, leucine and isoleucine biosynthesis	1.3	130.0860	1.3	0.6	0.043
N-acetyl-l-aspartate	65065	C_6_H_9_NO_5_	alanine, aspartate and glutamate metabolism	0.8	174.0397	0.0	2.3	0.010
l-glutamine	5961	C_5_H_10_N_2_O_3_	alanine, aspartate and glutamate metabolism	1.3	147.0763	0.4	1.3	0.023
N-acetylisatin	11321	C_10_H_7_NO_3_	tryptophan metabolism	9.8	190.0497	1.3	0.6	<0.001
5-methoxyindoleacetate	18986	C_11_H_11_NO_3_	tryptophan metabolism	4.7	206.0812	0.0	2.2	0.018
2-aminophenol	5801	C_6_H_7_NO	tryptophan metabolism	3.4	108.0441	3.2	0.7	0.026
(R)-(indol-3-yl)lactate	676158	C_11_H_11_NO_3_	tryptophan metabolism	5.1	204.0657	1.0	1.4	0.026
indoxyl	50591	C_8_H_7_NO	tryptophan metabolism	3.5	134.0599	1.4	1.8	0.038
6-hydroxykynurenate	440752	C_10_H_7_NO_4_	tryptophan metabolism	3.7	204.0293	0.9	2.1	0.015
leukotriene B4	5280492	C_20_H_32_O_4_	arachidonic acid metabolism	7.3	337.2368	1.9	0.8	0.027
leukotriene E4	5280879	C_23_H_37_NO_5_S	arachidonic acid metabolism	4.7	438.2293	3.5	0.4	0.029
20-HETE	5283157	C_20_H_32_O_3_	arachidonic acid metabolism	10.5	319.2273	1.8	0.8	0.033
arachidonic acid	444899	C_20_H_32_O_2_	arachidonic acid metabolism	10.2	305.2472	1.0	0.8	0.050

**Figure 3:**
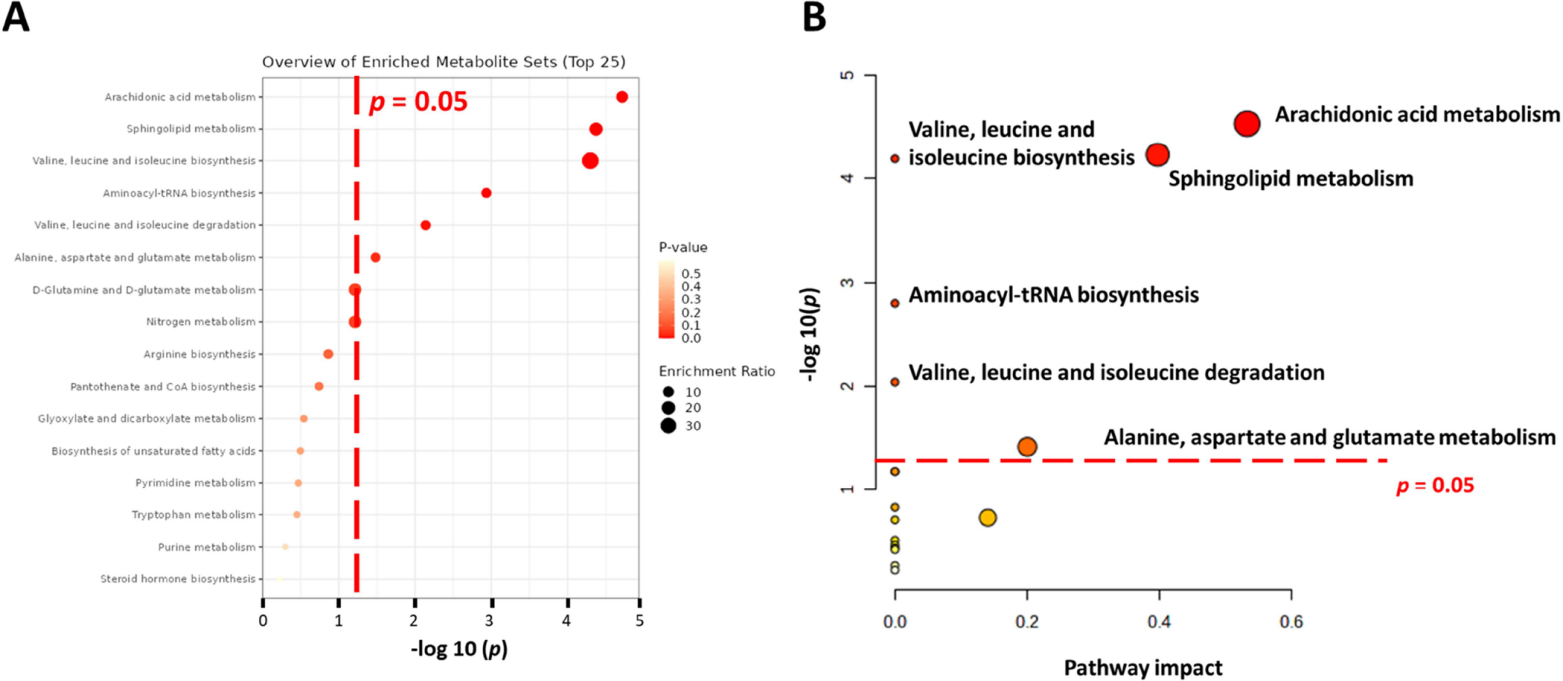
Identifying the key specific metabolic pathway for Alzheimer’s disease (AD). (A) *x*-axis displays the −log10 (*p*) from the enrichment analysis, and the size of the circles per metabolic pathway represents the enrichment ratio computed by observed hits/expected hits. The top six most significantly enriched pathways by enrichment analysis were ranked based on *p* as follows: arachidonic acid metabolism (*p* < 0.001), sphingolipid metabolism (*p* < 0.001), valine, leucine, and isoleucine biosynthesis (*p* < 0.001), aminoacyl-tRNA biosynthesis (*p* = 0.001), valine, leucine and isoleucine degradation (*p* = 0.007), and alanine, aspartate and glutamate metabolism (*p* = 0.033). (B) *x*-axis shows the calculation of pathway impact values, which is the accumulated percentage from the metabolic nodes of matched metabolites against the total pathway importance. The *y*-axis demonstrates −log10 (*p*) from the enrichment analysis. The pathway impact values suggested that the corresponding pathway is located at the key or independent positions of the metabolic network. The pathway impact values of the three key metabolic pathways are ranked as the following: arachidonic acid metabolism (impact = 0.52), sphingolipid metabolism (impact = 0.39), and alanine, aspartate, and glutamate metabolism (impact = 0.20).

A metabolite set enrichment analysis was employed using the MetaboAnalyst 5.0 online platform to identify the metabolic pathway related to the discriminatory metabolites discovered by the untargeted metabolomics approach.^25^ To evaluate the key perturbed metabolic pathways for the pathogenesis of AD, a total of 45 perturbed metabolites discovered by either untargeted or targeted metabolomics approaches were subjected to enrichment and pathway analysis. As depicted in Figure 3A, the *x*-axis displays the −log10 (*p*) from the enrichment analysis, and the size of the circles per metabolic pathway represents the enrichment ratio computed by observed hits/expected hits. The null hypothesis is that the correlation between the metabolite set of interest and the corresponding metabolic pathway is random. The top six most significantly enriched pathways by enrichment analysis were ranked based on *p* as follows: “arachidonic acid metabolism (*p* < 0.001)”, “sphingolipid metabolism (*p* < 0.001)”, “valine, leucine and isoleucine biosynthesis (*p* < 0.001)”, “aminoacyl-tRNA biosynthesis (*p* = 0.001)”, “valine, leucine and isoleucine degradation (*p* = 0.007)”, and “alanine, aspartate and glutamate metabolism (*p* = 0.033)”. It was represented that the six metabolic pathways were not random hits by enrichment analysis, indicating that they might contribute to AD pathogenesis. In Figure 3B, the *x*-axis shows the calculation of pathway impact values, which are the accumulated percentage from the metabolic nodes of matched metabolites against the total pathway importance. The pathway impact values suggest that the corresponding pathway is at the metabolic network’s key or independent positions. The pathways impact values of the six key metabolic pathways were ranked as the following: “arachidonic acid metabolism (impact = 0.52)”, “sphingolipid metabolism (impact = 0.39)”, “alanine, aspartate and glutamate metabolism (impact= 0.20)”, “valine, leucine and isoleucine biosynthesis (impact = 0.00)”, “aminoacyl-tRNA biosynthesis (impact = 0.00)”, and “valine, leucine and isoleucine degradation (impact = 0.00)”. The topological results indicated that the “valine, leucine, and isoleucine biosynthesis”, “aminoacyl-tRNA biosynthesis”, and “valine, leucine, and isoleucine degradation” were isolated positions of the metabolic network. Consequently, the three major metabolic pathways that contributed to perturbed metabolism in 6-month 5xFAD transgenic mice are summarized: “arachidonic acid metabolism”, “sphingolipid metabolism”, and “alanine, aspartate, and glutamate metabolism”.

### Investigation of Perturbed Metabolic Pathways in the Early Stage Using a 2-Month-Old 5xFAD Mice Model

The metabolic perturbations of 27 and 18 metabolites were discovered in the 6-month 5xFAD mice compared to WT mice by the untargeted and targeted metabolomics approach. Moreover, the three key perturbed metabolic pathways, including arachidonic acid metabolism, sphingolipid metabolism, and amino acid metabolism, were discovered in 6-month 5xFAD transgenic mice. To investigate whether the alternations of these metabolites and associated metabolic pathways were observed in the early stage of AD, the HRMS-based targeted metabolomics approach was performed in the 2-month 5xFAD mice model. Since the previous study indicated that 2-month 5xFAD transgenic mice do not have a Y-maze deficit and detected starting of the earliest accumulations of amyloid plaques, the 2-month 5xFAD animal model was selected to investigate the perturbations of metabolome for the early stage of AD.^5^ In the 2-month animal model, the 20 hair samples were collected from ten 2-month 5xFAD and ten 2-month WT mice and then subjected to sample preparation. Subsequently, the hair extracts were subjected to an HRMS-based targeted analysis. The 27 discriminatory metabolites discovered by the untargeted metabolomics approach and 150 metabolites associated with the key metabolic pathways were used to perform the HRMS-based targeted metabolomics approach, respectively, of which 138 chemical compounds were identified in the hair metabolome of two-month 5xFAD mice. Among these, six metabolites, namely, sphinganine, 3-dehydrosphinganine, sphingosine, 11,12-DHET, prostaglandin F2alpha, and N-acetylaspartylglutamate were found significantly different in 2-month 5xFAD mice compared to WT. These results indicated that arachidonic acid and sphingolipid metabolism might be associated with the early stage of AD pathogenesis (Table 2).

**Table 2:** Discriminatory Metabolites Identified in the 2-Month 5xFAD Transgenic Mice

metabolite name	PubChem CID	chemical formula	metabolic pathway	retention time (min)	measured *m*/*z*	mass accuracy (ppm)	fold change	*p* values
sphinganine	91486	C_18_H_39_NO_2_	sphingolipid metabolism	9.0	302.3050	1.3	0.5	0.003
3-dehydrosphinganine	439853	C_18_H_37_NO_2_	sphingolipid metabolism	0.6	300.2892	1.7	1.5	0.028
sphingosine	5280335	C_18_H_37_NO_2_	sphingolipid metabolism	8.8	300.2893	1.5	0.7	0.038
11,12-DHET	5283146	C_20_H_34_O_4_	arachidonic acid metabolism	9.1	339.2525	1.5	1.1	0.026
prostaglandin F2alpha	5280363	C_20_H_34_O_5_	arachidonic acid metabolism	8.6	355.2475	1.0	1.6	0.040
N-acetylaspartylglutamate	188803	C_11_H_16_N_2_O_8_	alanine, aspartate and glutamate metabolism	2.7	303.0826	1.1	1.9	0.017

### Evaluation of Potential AD Biomarker Candidates in Human Subjects

The distinguishing performances of 45 biomarker candidates discovered in the 5xFAD mice model by both untargeted and targeted metabolomics approaches were further evaluated in an additional independent cohort of hair samples from 10 patients with AD and 10 cognitively healthy controls. The clinical characteristics are demonstrated in Table 3, and the detailed information for each participant is shown in Table S6. The *t* test assessed significant differences in continuous variables, including age, MoCA scores, and body mass index (BMI). The age (*p* = 0.15) and BMI (*p* = 0.72) were not significantly different between patients with AD and controls. The Chi-square test was also employed to evaluate significant differences in discrete variables, including family medical history, cosmetic hair treatment, smoking habits, and alcohol intake. The family medical history (*p* = 0.53), cosmetic hair treatment (*p* = 0.88), smoking habits (*p* = 0.53), and alcohol intake (*p* = 0.30) did not demonstrate significant differences between these two groups.

**Table 3:** Clinical Characteristics of Patients with AD and Normal Cognitive Function Subjects^a^

characteristics		AD	HC	*p*-value^b^
sex	male	3	3	1.00
	female	7	7	
age		74.5 ± 8.1	68.9 ± 8.7	0.15
body mass index (kg/m^2^)		24.1 ± 4.2	23.5 ± 2.9	0.72
MoCA score		14.5 ± 7.8	28.0 ± 1.6	<0.01
family medical history	yes	2	1	0.53
	no	8	9	
cosmetic hair treatment	never	5	5	0.88
	perming	2	1	
	dyeing	2	2	
	perming and dyeing	1	2	
smoking habits	past	2	1	0.53
	no	8	9	
alcohol intake	past	0	1	0.30
	no	10	9	

a MoCA: Montreal cognitive assessment.

b The *t* test was employed for the continuous variables, including age, BMI, and MoCA score, between AD and controls. The chi-square test was conducted for discrete variables, containing family medical history, cosmetic hair treatment, smoking habits, and alcohol intake.

**Figure 4:**
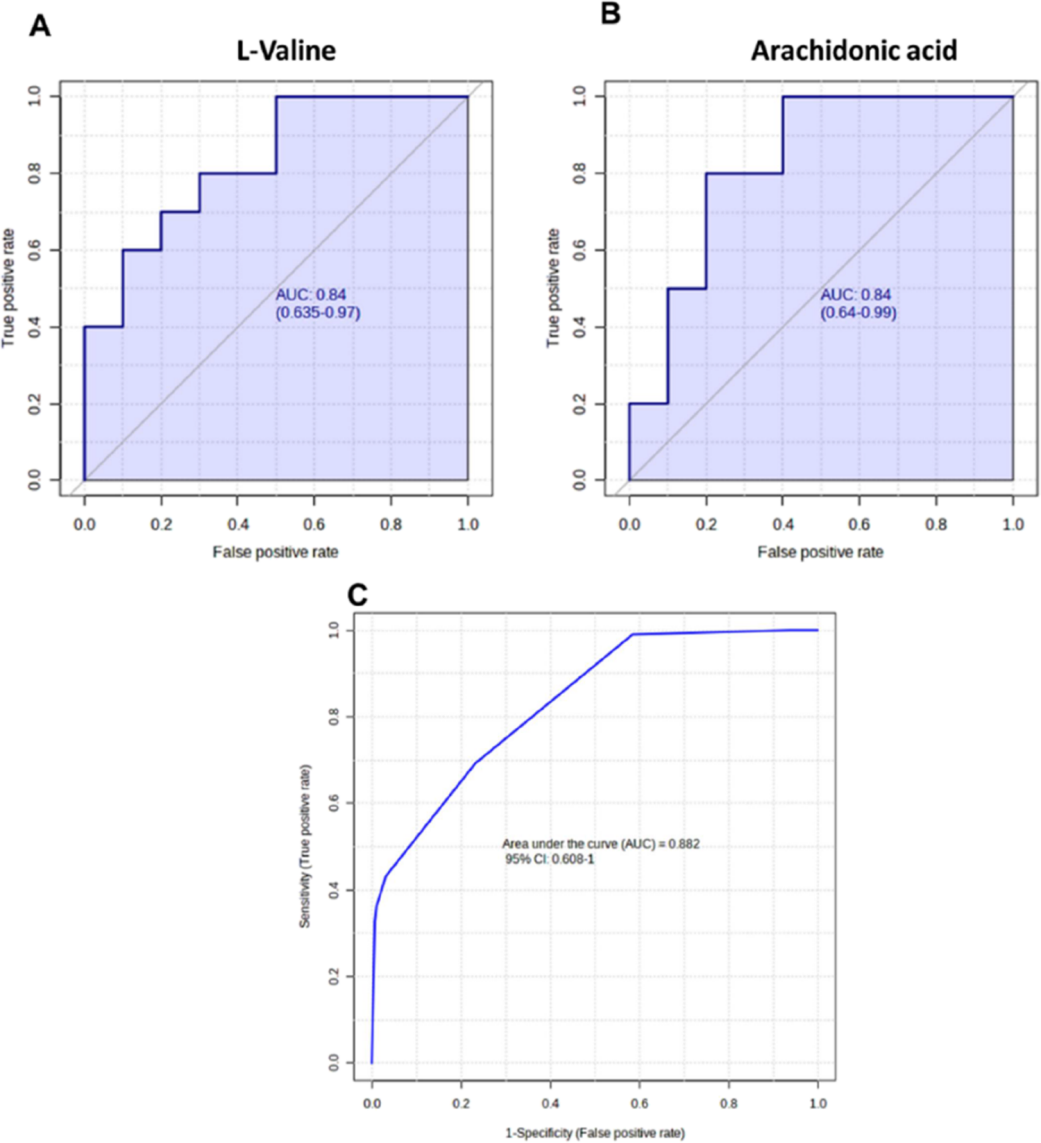
ROC curve of the two metabolites and a composite panel in human subjects. The AUC values of both (A) l-valine and (B) arachidonic acid were 0.84. (C) Using a composite panel of these two biomarker candidates, the AUC value achieved 0.88 (95% CI: 0.61–1.00) for distinguishing patients with AD from healthy controls. Diagnostic sensitivity and specificity of the composite panel in AD patients were 80 and 70%, respectively.

The receiver operating characteristic (ROC) curves of the two metabolites, l-valine and arachidonic acid, between AD patients and controls in the validation set are plotted in Figure 4A,B. The area under these two metabolites’ curve (AUC) values were 0.84. These chemical compounds had at least 80% sensitivity and 80% specificity. When a composite panel of these two metabolites was used, the AUC value achieved 0.88 (95% CI: 0.61–1.00) for distinguishing patients with AD from healthy controls (Figure 4C). Diagnostic sensitivity and specificity of the composite panel in AD patients were 80% and 70%, respectively, suggesting that a panel of these two metabolites discovered in the 5XFAD mice model might be used for distinguishing patients with AD from healthy subjects. These two metabolites, l-valine and arachidonic acid, may play a significant role in the pathogenesis of AD and serve as biomarkers for its prevention, as their changes might precede disease phenotypes over decades. The correlation analysis between the levels of these two altered metabolites and MoCA scores was conducted, as shown in Figure S2. The correlation coefficient values were 0.44 (*p* = 0.05) for l-valine and −0.50 (*p* = 0.03) for arachidonic acid. The statistically significant correlation coefficients indicate a noteworthy association, where higher levels of l-valine and lower levels of arachidonic acid may relate to the impairment of cognitive function. This finding indicated that the combination of these two metabolites might be used as an early indicator of AD diagnosis and prevention. Further research is necessary to explore the mechanistic link between these metabolites and AD progression, which could open new avenues for early intervention strategies in AD.

## Discussion

This study employed untargeted and targeted metabolomics approaches to discover the alternations of 45 differential metabolites and the three key corresponding metabolic pathways using hair metabolome from 6-month 5xFAD and WT mice. The metabolic perturbations between the 2- and 6-month transgenic mice were investigated. Since Aβ plaque deposition in the brain tissue can occur several decades before the onset of AD symptoms, we performed a 2-month-old mice model to characterize the perturbations of metabolic pathways for early AD.^26^ Subsequently, we revealed that the three metabolic pathways, arachidonic acid, sphingolipid, and alanine metabolism, might be involved in the early pathogenesis of AD. Additionally, we evaluated the distinguished performance of the discovered metabolites between 10 patients with AD and 10 control subjects, resulting in l-valine and arachidonic acid as potential biomarkers for AD.

Compared to conventional biological specimens, such as blood and urine, hair reflects the endogenous chemical composition of the body burden over several months to years since its growth rate was approximately 1 cm per month.^13^ Circulating metabolites might enter and be incorporated into the hair matrix through passive diffusion during its formation, reflecting the circulating chemical composition.^13,19,27^ As the cells of hair follicles die and fuse to generate hair strands, the chemical compounds are retained and accumulate in this highly stable structure. The hair biospecimen has recently been used to monitor the chemical exposome by untargeted and suspect screening approaches.^28,29^ Moreover, previous studies indicated that hair was used to discover biomarkers associated with pregnancy complications.^9,10^ Therefore, it was reported that hair could serve as an emerging matrix for biomonitoring investigation and biomarker discovery, and the sequential alternations of the hair metabolome in a specific time can be monitored and observed from segmental hair analysis.^10^

**Figure 5:**
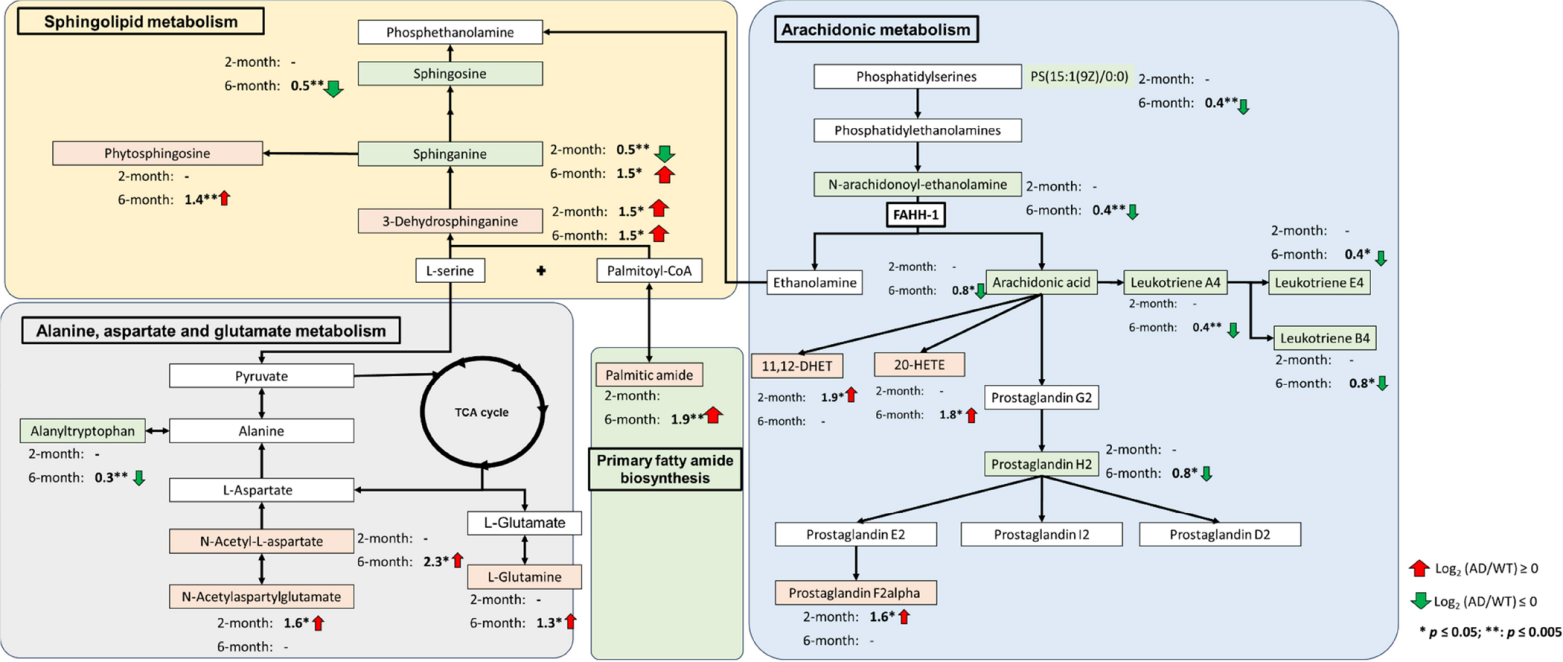
Summary of the altered metabolites and the key perturbed pathways in 5XFAD mice. The metabolic pathways retrieved based on the KEGG database and from the literature were used to plot and summarize the perturbed vital metabolic pathways for AD. The three key metabolic pathways, including arachidonic acid metabolism, sphingolipid metabolism, and alanine, aspartate, and glutamate metabolism, discovered in hair might contribute to the pathogenesis of AD.

The metabolic pathways retrieved based on the KEGG database and from the literature were used to plot and summarize the perturbed key metabolic pathways for AD in Figure 5.^30−32^ The N-arachidonoyl-ethanolamine, anandamide (AEA), is an endocannabinoid implicated in numerous physiological processes, such as cognition, inflammatory pain, and inflammation.^30^ AEA is released by hydrolysis of N-arachidonoyl-substituted phosphatidylethanolamine species generated from the transfer of arachidonic acid to phosphatidylethanolamine (PE). A previous study has reported that the levels of anandamide are lowered in the midfrontal cortex and temporal cortex of AD patients compared to controls.^33^ Our observation indicated that the concentration of AEA, an endocannabinoid, was significantly lower in 5xFAD transgenic mice compared with WT mice. This finding is consistent with the previous study and suggests that during the etiology of the 5xFAD model, AEA might be subject to altered regulation or increased degradation.^34^ This observed change in AEA metabolism in the 5xFAD model underscores the importance of understanding how changes in bioactive lipid signaling molecules may contribute to the pathogenesis of AD. One such bioactive lipid signaling molecule of interest is palmitic amide, a primary fatty acid amide. Palmitic amide is formed by cleavage of palmitic-CoA. Because primary fatty acid amides are structurally similar to AEA, there might be interaction with CB1 receptors, which are endocannabinoid receptors, representing that they compete with endocannabinoids to bind the active site of the receptor.^31^ In our observations, the decreased level of AEA and elevated level of palmitic amide were found in the 6-month-old 5xFAD transgenic mice model, indicating that monitoring the balance of primary fatty acid amide and endocannabinoid in the biological system might reflect the risk of AD. Therefore, these findings unveiled that a chronic deficiency in endocannabinoids might play a role in the pathogenesis of AD.

The fatty acid amide hydrolase-1 (FAHH-1) was performed to degrade AEA into arachidonic acid (AA) and ethanolamine, so the metabolites of AA metabolism were characterized by targeted analysis.^30^ In this study, leukotriene B4 (LTB4), leukotriene A4 (LTA4), leukotriene E4 (LTE4), prostaglandin H2 (PGH_2_), prostaglandin F2α (PGF2α), 20-HETE, and 11,12-DHET were identified by either targeted or untargeted metabolomic analysis. PGF2α and 11,12-DHET were upregulated in 2-month-old 5XFAD transgenic mice compared with WT mice. Results in this study are consistent with our previous observation, which was performed in the Aβ-injected rat model, suggesting that the use of hair could reflect the perturbations in AA metabolism influenced by the production of Aβ and accumulation of amyloid plaques in the brain tissues.^35^ These findings suggest that the AA metabolism, which participates in cellular signaling as a second messenger and a key inflammatory intermediate, may be associated with neurological toxicity generated by Aβ accumulations.

Sphingolipid metabolism is a significant metabolic pathway discovered in a 6-month-old 5xFAD transgenic mice model. It has been demonstrated that this pathway is influenced during the early onset of AD. Sphingolipids are bioactive molecules that regulate cell survival, cellular stress, and cell death.^36^ Additionally, they served as secondary messengers in health and disease.^36^ The present study characterized 3-dehydrosphinganine, sphinganine, N-acetylsphinganine, sphingosine, and phytosphingosine. In the previous study, the levels of sphinganine and phytosphingosine in the plasma samples of AD patients were both found to be lower than those in the controls.^37^ This result in the previous study contrasts with our observation of overexpression of both sphinganine and phytosphingosine in the hair of 6-month-old 5xFAD mice. The possible reason for these two metabolites circulating in the blood may be their incorporation and subsequent accumulation in the hair sample of mice before the production and accumulation of Aβ in 5xFAD mice. Significant changes in 3-dehydrosphinganine and sphinganine were discovered in both 2-month-old and 6-month-old 5xFAD transgenic mice compared to those in WT mice.

Among the 45 altered metabolites associated with AD identified by the animal model, valine and arachidonic acids demonstrated high performance in distinguishing patients with AD from control subjects. A positive correlation was observed between the levels of l-valine and MoCA scores in our study, corroborating findings from a previous study.^38^ This study indicates that reduced levels of valine in plasma might contribute to cognitive decline, while higher concentrations could be associated with a decreased risk of developing AD.^38,39^ Our observations suggest that the concentrations of l-valine in hair may mirror its variations in plasma. Valine, a branched-chain amino acid with an aliphatic side chain, can easily cross the blood-brain barrier and is converted into glutamate, an excitatory neurotransmitter in the brain.^40,41^

Moreover, a previous study reported higher levels of valine in CSF of patients with AD than in mild cognitive impairment (MCI) subjects, indicating that this metabolite could serve as a biomarker to monitor the progression of dementia.^39,42^ Besides, the decreased circulating levels of other branched-chain amino acids, leucine, and isoleucine are associated with preclinical dementia and MCI in human subjects.^43^ Our findings suggested that the animal model associated the branched-chain amino acids with the development of AD. Therefore, the changes in branched-chain amino acids in hair might reflect their variation in the bloodstream. In addition to valine, our findings indicate a negative correlation between the levels of arachidonic acid and MoCA scores. This result indicated that the elevated levels of arachidonic acid might be associated with cognitive function impairments. The previous studies revealed that patients with AD in the Tunisian population had notably relatively high levels of arachidonic acid in their plasma compared to controls.^44^

Additionally, an interaction between the apolipoprotein E phenotype and the high arachidonic acid/docosahexaenoic acid ratios contributed to the pathogenesis of AD.^45^ The possible reason was that excessive levels of arachidonic acid might link to neuroinflammation and neuronal damage in the brain, contributing to the pathogenesis of AD.^46^ The arachidonic acid is significantly enriched in the brain. The arachidonic acid derivatives, prostaglandins, and leukotrienes, are important in the pathogenesis of neurodegenerative diseases, such as AD or Parkinson’s disease.^46^

It is important to consider some inherent limitations that may affect the interpretation of our findings in this study. First, the Shapiro-Wilk test was applied to evaluate the normality, while the normality tests lacked the power to detect non-normal distribution in small size.^47^ Consequently, the features identified in the HRMS data set could be more accurately discerned with increased sample size. Second, the AD patients were diagnosed only based on evaluation of the behavioral symptoms, including memory impairment and cognitive examination, by professional neurological physicians. However, it is noted that the initial stages of AD often manifest symptoms similar to those observed in other types of dementia.^26^ Although analyzing APOE genotype, measuring Aβ and tau in the blood or CSF, and employing imaging techniques provide valuable information for diagnosing AD, the complex sampling procedure and high cost associated with these methods present practical clinical challenges. Third, hair samples with cosmetic treatment collected from human subjects were included in this study. The use of cosmetic hair treatments, such as perming and dying, might be a confounding factor for evaluating the altered metabolites for distinguishing patients with AD from controls since it has been indicated that cosmetic treatment might alter the chemical composition of human hair.^48^ For example, Eisenbeiss and colleagues suggested that some metabolites, such as amino acids, purines, nucleosides, and carnitines, might be influenced by their levels of oxidative blenching. Ideally, the hair sample with cosmetic treatments should be excluded, while recruiting participants for biomarker discovery poses challenges.

The 5xFAD transgenic mice model, which exhibits extracellular Aβ accumulation and senile plaques, was employed in this study. As an important tool for understanding the pathogenesis of AD, many animal models are proposed.^49^ These discriminatory chemicals should be validated and evaluated in other animal models, such as a triple transgenic AD mouse model expressing amyloid plaques and neurofibrillary tangles, to confirm the possible biomarkers for AD pathogenesis. The clinical diagnostic accuracy of the discriminatory chemicals discovered from the hair samples from the transgenic mice model for patients with AD requires further investigation and validation. If the discovered discriminatory chemicals in the transgenic mice model are validated and evaluated in an additional large number of AD patients, these chemicals offer a promising way to diagnose AD in a noninvasive manner.

## Conclusions

In this study, we used HRMS-based untargeted and targeted metabolomics to characterize the metabolic alterations in the hair of a 5xFAD transgenic mice model. Forty-six metabolites are identified by either untargeted or targeted metabolomic analysis, of which 27 were discovered by untargeted and 19 were discovered by targeted metabolomics approach. Our results indicated that arachidonic acid metabolism, sphingolipid metabolism, alanine, aspartate, and glutamate metabolism were the key perturbed pathways in the 5xFAD mice model, as depicted in Figure 5. Moreover, six metabolites associated with these three metabolic pathways were found to be related to the early onset of AD. This outcome shows that hair can be a biospecimen to detect the alternations of the three metabolic pathways, and the six metabolites might be used as biomarker candidates for early AD detection.

## Materials and Methods

### Animal Experiments

The study utilized 10 heterozygous 5xFAD transgenic mice (B6SJL-Tg(APPSwFlLon;PSEN1M146LL286 V)6799Vas/Mmjax) and 10 wild-type littermates. These transgenic mice carried five genes that accelerated the deposition of amyloid plaques in the brain. The mice were kept in a controlled environment with a stable temperature of 23–25 °C and a 12 h light/dark cycle. They had unrestricted access to food and water. The hair samples from 2- and 6-month-old mice were collected, and these time points corresponded to stages before the onset of amyloidosis and during significant accumulation of amyloid plaques, respectively. The experimental protocols and procedures conducted on the animals were approved by the International Animal Care and Use Committee (IACUC #109338).

### Participant Collection

Twenty participants aged older than 40 years and with no stroke history or known malignancy, including 10 patients with AD and 10 controls, were recruited from the National Cheng Kung University Hospital, Tainan, Taiwan. The MoCA was administrated to assess the cognitive performance of the participants. The control subjects (*n* = 10) underwent the MoCA examination and had a MoCA score of ≥26 points. All participants in this study signed a written informed consent form according to the rules and requirements of the Institutional Review Board of National Cheng Kung University Hospital (IRB approval no. B-ER-108-188).

### Mice and Human Hair Sample Collection and Preparation

At two distinct ages, 2 and 6 months old, mouse hair at the back was shaved, and the samples were stored in brown glass tubes at 4 °C. Hair samples from human subjects were collected by cutting approximately 0.5 cm away from the scalp with scissors. After collecting the hair samples, the 3-cm segments were measured from the end point of the hair cut from the scalp. These 3 cm hair samples from human subjects were secured in aluminum foil and stored at 4 °C.

### Sample Preparation

Both mice and human hair samples underwent the same sample preparation procedure. Since the chemicals retained on the hair surface might infer the characterization of the metabolome, the decontamination procedure was performed according to the Society of Hair Testing guideline for removing contaminants.^22^ Based on this guideline, acetone and deionized water were recommended for decontamination. A 30-mg hair sample was washed with 1.8 mL of acetone (HPLC grade, from JT Baker), followed by washing with 1.8 mL of deionized water (milli-Q system, Merck). Both processes were carried out using an ultrasonic bath for 2 min. The solvents for decontamination in the tube were discarded, and the hair sample was dried through evaporation by N_2_ for approximately 90 min. The dried hair samples were cut into 0.2 cm pieces and subjected to the extraction procedure as described.^21^ The hair sample was mixed with 1.5 mL of methanol (LC–MS grade, from JT baker)/phosphate-buffered saline (ACS grade, from Sigma-Aldrich) (50/50, v/v) and sonicated for 4 h at 55 °C. The extracts were centrifuged at 20,000 × *g* for 15 min, and the supernatants were collected. The supernatant was dried by a speed vacuum (Eyela CVE-2200) overnight, and the residue was reconstituted with 150 μL of 50% methanol in deionized water. A quality control (QC) sample was prepared by pooling the hair samples from an aliquot of the hair samples and subjected to the same extraction procedure. A QC sample was prepared parallel to study samples and analyzed after the 4 sample injections. For blank signal assessment, the extraction solutions (methanol/phosphate-buffered saline 50/50, v/v) in duplicate were subjected to the same sample preparation procedure. The minimum peak height for data processing was set at 10,000, based on the average abundance of signals with an average signal-to-noise ratio below 3 in the solvent blank.

### Ultrahigh Performance Liquid Chromatography–High Resolution Mass Spectrometry Analysis

An Ultimate 3000 system and a Q Exactive Orbitrap HRMS system (Thermo Fisher Scientific) were used for hair sample analysis. Chromatographic separation was performed by a Luna C18 column (2.1 × 100 mm, 2.0 μm, purchased from Phenomenex) maintained at 40 °C. The mobile phase consisted of acetonitrile (LC–MS grade, from JT Baker)/deionized water (2/98) with 0.1% formic acid (ACS grade, from Sigma-Aldrich) as mobile phase A, while mobile phase B consisted of 100% acetonitrile with 0.1% formic acid. The mobile phase flow rate was set at 250 μL/min, and the linear elution gradient was as follows: 2% B for 1 min; 2–99% B for 10 min; 99% B for 2 min; and 2% B for 1 min. Each sample was injected with a volume of 5 μL.

The Q Exactive Orbitrap was operated in positive and negative ionization modes to acquire the HRMS data. The mass range for the full scan method was set from *m*/*z* 100–1000, with a resolution of 70,000. Discriminatory features underwent parallel reaction monitoring (PRM) analysis in MS/MS mode. The MS/MS analysis was conducted using higher-energy collisional dissociation (HCD) at a resolution of 17,500. The normalization collision energy was set at 30 and 50%, respectively, with the same chromatographic conditions.

### Data Processing, Statistical Analysis, Chemical Structure Identification, and Pathway Analysis

The raw data obtained from the analysis were processed by MS-DIAL 4.70, which performed peak detection and alignment.^50^ To convert the raw data into the mzML file format with the centroid data type, MSConvert (Version: 3.0.22175-93442d4) was utilized.^51^ The converted files were then imported into MS-DIAL 4.70 for further data processing using the following parameters: minimum peak height of 10,000, minimum peak width of 10 scans, retention time tolerance of 0.2 min, and mass tolerance of 0.0015 μ. Subsequently, an alignment peak table containing accurate masses, retention times, and peak abundances was exported.

To enable comparability between samples, each raw abundance was normalized by dividing it by the sum of the raw abundances of all peaks in the corresponding sample before conducting multivariate and univariate statistical analysis. PCA, a multivariate statistical method, was used to evaluate the reproducibility of the analytical method. The PCA score plots were employed to explain the variance within the HRMS data set by the PCs, which are mutually uncorrelated. The analysis was conducted in R version 4.3.0 and plotted using the package “ggplot2” in R 4.3.0. The normality of each feature in the HRMS data set for these two groups was assessed using the Shapiro–Wilk test by R 4.3.0. According to the normality test results, either the Student *t* test or the Mann–Whitney *U* test were employed to discover the discriminatory metabolites using R version 4.3.0 and RStudio (2023.06.0 + 421). Discriminatory features were evaluated by detecting all features that demonstrated a fold change ≥1.2 or ≤0.8 and *p* ≤ 0.005. The fold change was calculated by dividing the average normalized abundance in 5xFAD mice by that in WT mice, and *p* was calculated using two-tailed unpaired Student *t* tests. The fold change and *p* values were calcuated in R 4.3.0, and the volcano plots were plotted using the package “ggplot2” in R 4.3.0.

In the targeted metabolomics approach, the “webchem” package in R 4.3.0 retrieved the chemical formula, monoisotopic mass, and InChIKey for all compounds in the metabolic pathways.^52^ These theoretical *m*/*z* values of the chemicals were computed based on their corresponding monoisotopic mass and molecular ion adduct using R 4.3.0. These theoretical *m*/*z* values were then compared to the experimental *m*/*z* values within a tolerance of 5 ppm, and annotations were made to the features based on the matches. A two-tailed unpaired Student *t* test was used to calculate the *p* of the identified features. The discriminatory features were evaluated based on the *p* ≤ 0.05 between 5xFAD and WT mice.

The identification of the chemical structures of the metabolites was accomplished through two approaches. Spectral database matching was employed by annotating metabolites using MS-DIAL 4.70. It involved comparing accurate mass and MS/MS spectra with those in the MassBank of North America (MoNA), downloaded on April 26, 2023, and LipidBlast on June 1, 2023,.^53^ The search windows were a relative mass tolerance of 5 ppm for the precursor ion *m*/*z* and a cutoff value of 70% from MS-DIAL for MS/MS spectral matching. An *in silico* approach was utilized with the formula finder and structure finder in MS-FINDER 3.52.^54^ The following search windows were employed: a relative mass tolerance of 5 ppm for the precursor ion *m*/*z* and a cutoff value 7.0 in the structure finder within MS-FINDER 3.52. This approach allowed for the prediction of metabolite chemical structures based on mass spectral data.

Pathway analysis was performed by MetaboAnalyst 5.0.^25^ This analysis included enrichment analysis and topological analysis. The enrichment analysis helped identify overrepresented metabolic pathways, while topological analysis assessed the overall impact of metabolites within the metabolic network. The hypergeometric test conducted the enrichment method, and topology analysis was used for relative-betweenness centrality. The KEGG database in MetaboloAnalyst 5.0 was used as the backend knowledge base.^55^

To evaluate the predictive performance of the model, ROC curves were plotted using MetaboAnalyst 5.0. Random forest analysis was also performed to generate a metabolite panel for predictive purposes.^25^ These analyses aid in assessing the diagnostic or predictive capability of the identified metabolites in the context of the studied metabolic pathways.

## Supporting Information Available

Results of multivariate statistical analyses on hair samples from three groups (Figure S1); results of a correlation analysis examining the relationship between the abundance of discriminatory metabolites and MoCA scores within a cohort of 20 participants (Figure S2); HRMS data sets obtained from 6-month-old 5xFAD mice, which were operated in both positive and negative ion modes (Table S1 and S2); discriminatory metabolites discovered by untargeted metabolomics (Table S3); results of an enrichment analysis carried out on the 27 discriminatory chemicals identified through the untargeted metabolomics approach (Table S4); compilation of metabolites associated with relevant metabolic pathways (Table S5); and detailed demographic information for each of the 20 study participants (Table S6) (ZIP)
