# Supplementary material for: Characterization of Hair Metabolome in 5xFAD Mice and Patients with Alzheimer’s Disease Using Mass Spectrometry-Based Metabolomics
Source: ACS Chem Neurosci. 2024 Jan 25;15(3):527–38. doi: 10.1021/acschemneuro.3c00587 (PMC10853927; doi:10.1021/acschemneuro.3c00587)
Supplement: Supplementary file 1 — cn3c00587_si_001.zip [file cn3c00587_si_001.zip › Supporting information_Revision.docx]

**Supporting information**

**Characterization of Hair Metabolome in 5xFAD Mice and Patients with Alzheimer’s Disease Using Mass Spectrometry-Based Metabolomics**

Chih-Wei Chang ^a,†^, Jen-Yi Hsu ^a,†^, Yu-Tai Lo ^b,c^, Yu-Hsuan Liu ^a^,
Onanong Mee-inta ^d^, Hsueh-Te Lee ^f^, Yu-Min Kuo ^d,e,*^, and Pao-Chi Liao ^a,g,^*

1. Department of Environmental and Occupational Health, College of Medicine, National Cheng Kung University, Tainan, 704, Taiwan
2. Department of Geriatrics and Gerontology, National Cheng Kung University Hospital, College of Medicine, National Cheng Kung University, Tainan, Taiwan.
3. Department of Public Health, College of Medicine, National Cheng Kung University, Tainan, Taiwan.
4. Institute of Basic Medical Sciences, College of Medicine, National Cheng Kung University, Tainan, 701, Taiwan
5. Department of Cell Biology and Anatomy, College of Medicine, National Cheng Kung University, Tainan, 701, Taiwan
6. Institute of Anatomy and Cell Biology, School of Medicine, National Yang Ming Chiao Tung University, Taipei, Taiwan
7. Department of Food Safety/Hygiene and Risk Management, College of Medicine, National Cheng Kung University, Tainan, 701, Taiwan

† Chih-Wei Chang and Jen-Yi Hsu have contributed equally to this work.

*Co-Corresponding authors:

Dr. Yu-Min Kuo

Department of Cell Biology and Anatomy, College of Medicine, National Cheng Kung University, Tainan, 701, Taiwan

Tel: 886-6-2353535 ext 5294

Fax: 886-6-2093007

Email: kuoym@mail.ncku.edu.tw

Dr. Pao-Chi Liao

Department of Environmental and Occupational Health, College of Medicine, National Cheng Kung University, 138 Sheng-Li Road, Tainan, 704, Taiwan,

Tel: 886-6-2353535 ext. 5566

Fax: 886-6-2752484

Email: [liaopc@mail.ncku.edu.tw](mailto:liaopc@mail.ncku.edu.tw)

**Figure Legend**

**Figure S1. Multivariate statistical analyses of hair samples in 5xFAD vs WT mice vs QC groups.** Principal component analysis (PCA), a multivariate statistical method, was used to evaluate the reproducibility of the analytical method. The PCA score plots were employed to explain the variance within the HRMS dataset by the principal components (PCs), which are mutually uncorrelated. The analysis was conducted in R 4.3.0 and plotted using the package "ggplot2" in R 4.3.0. Comparable patterns of samples are tightly clustered in the score plots. The two plots of the HRMS dataset obtained from positive and negative ion modes revealed clear and tight clusters of 5 QC samples, suggesting minimal technical errors occurred in this research. However, the PCA score plots did not clearly distinguish between AD transgenic and WT mice. **A.** In the HRMS dataset obtained from positive ion mode, PC1 and PC2 explained 29.3% and 25.1% of the total variance, respectively. **B.** In the aligned features from negative ion mode, PC1 and PC2 accounted for 44.8% and 13.5% of the total variance, respectively.

**Figure S2. The correlation analysis of normalized abundance and MoCA scores within 20 participants (10 patients with AD and 10 controls).** The Pearson correlation analysis between normalized abundance of metabolites and MoCA scores was conducted by R 4.3.0. The correlation coefficient values were 0.44 (p = 0.05) and -0.50 (p = 0.03) for **A.** L-valine and **B.** arachidonic acids, respectively. The statistically significant correlation coefficients indicate a noteworthy association, where higher levels of L-valine and lower levels of arachidonic acid may relate to the impairment of cognitive function. This finding indicated that the combination of these two metabolites might be used as an early indicator in AD diagnosis and prevention.

**Table Legends**

**Table S1. The HRMS dataset of 6-month-old 5xFAD model operated in positive ion mode.** The dataset was generated by MS-DIAL 4.70 by performing peak detection and peak alignment. A total of 26,161 aligned features was detected in the positive ion modes.

**Table S2. The HRMS dataset of 6-month-old 5xFAD model operated in negative ion mode.** The dataset was generated by MS-DIAL 4.70 by performing peak detection and peak alignment. A total of 19,187 aligned features was detected in the negative ion mode.

**Table S3. Discriminatory metabolites discovered by untargeted metabolomics.** Among 155 discriminatory features of which fold change ≥ 1.2 or ≤ 0.8 with p value below 0.005, 27 discriminatory metabolites were successfully identified their chemical structures. The discriminatory metabolites were identified using matching experimental MS/MS spectrum obtained from fragmentation analysis to that retrieved from mass spectral database or predicted from *in silico* approach.

**Table S4. Enrichment analysis of 27 discriminatory chemicals discovered by untargeted metabolomics approach.** Among the 27 discriminatory metabolites, 10 were included in the KEGG database, of which 8 metabolites were identified to 7 relevant metabolic pathways by MetaboAnalyst. The most significant pathways were ‘Sphingolipid metabolism’ (*p* = 0.002) and ‘Valine, leucine and isoleucine biosynthesis’ (*p* = 0.025).

**Table S5. Metabolites in associated metabolic pathways for targeted metabolomics analysis.** A total of 241 chemicals are included in the seven relevant metabolic pathways. Among these, two metabolites were included in N-(icosanoyl)ethanolamine metabolism by the previous study^1^, and 239 metabolites were included in the six pathways in the KEGG database.

**Table S6. The detail clinical characteristics for each participant.** A total of 20 participants, including 10 patients with AD and 10 non-dementia subjects, was collected in this study. The individual information was collected by the questionnaire and signed a written informed consent form according to the rules and requirements of the Institutional Review Board of National Cheng Kung University Hospital (IRB approval no. B-ER-108-188)

**
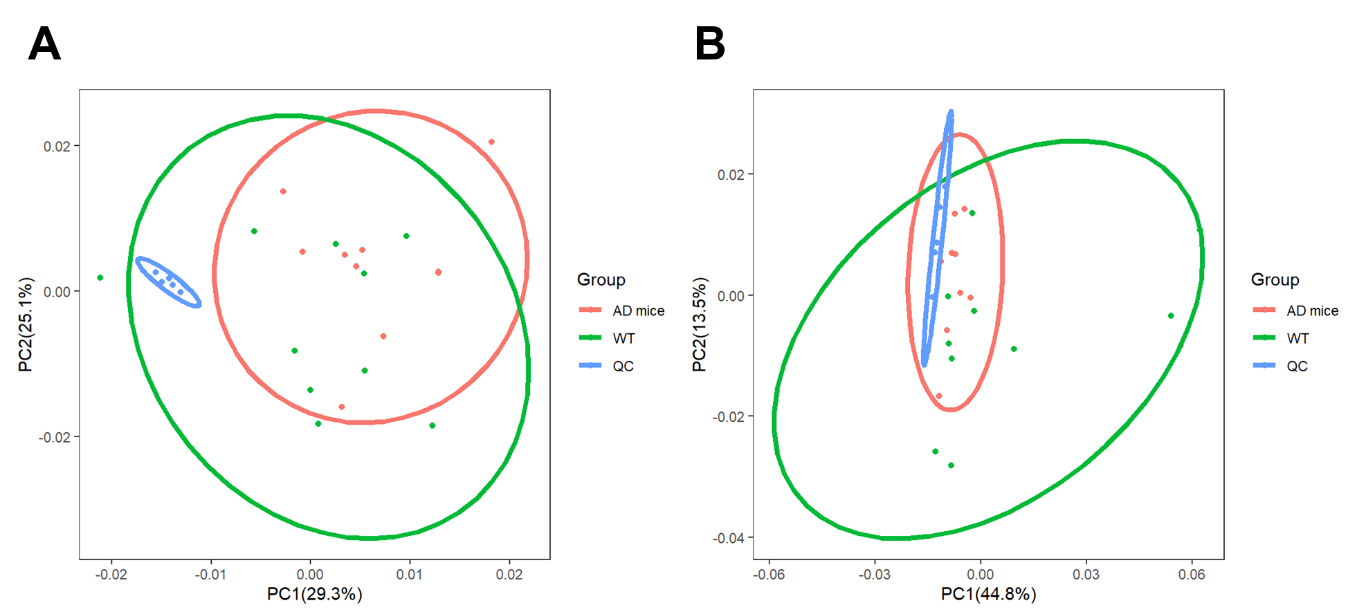
**

**Figure S1. Multivariate statistical analyses of hair samples in 5xFAD vs WT mice vs QC groups.** Principal component analysis (PCA), a multivariate statistical method, was used to evaluate the reproducibility of the analytical method. The PCA score plots were employed to explain the variance within the HRMS dataset by the principal components (PCs), which are mutually uncorrelated. The analysis was conducted in R 4.3.0 and plotted using the package "ggplot2" in R 4.3.0. Comparable patterns of samples are tightly clustered in the score plots. The two plots of the HRMS dataset obtained from positive and negative ion modes revealed clear and tight clusters of 5 QC samples, suggesting minimal technical errors occurred in this research. However, the PCA score plots did not clearly distinguish between AD transgenic and WT mice. **A.** In the HRMS dataset obtained from positive ion mode, PC1 and PC2 explained 29.3% and 25.1% of the total variance, respectively. **B.** In the aligned features from negative ion mode, PC1 and PC2 accounted for 44.8% and 13.5% of the total variance, respectively.


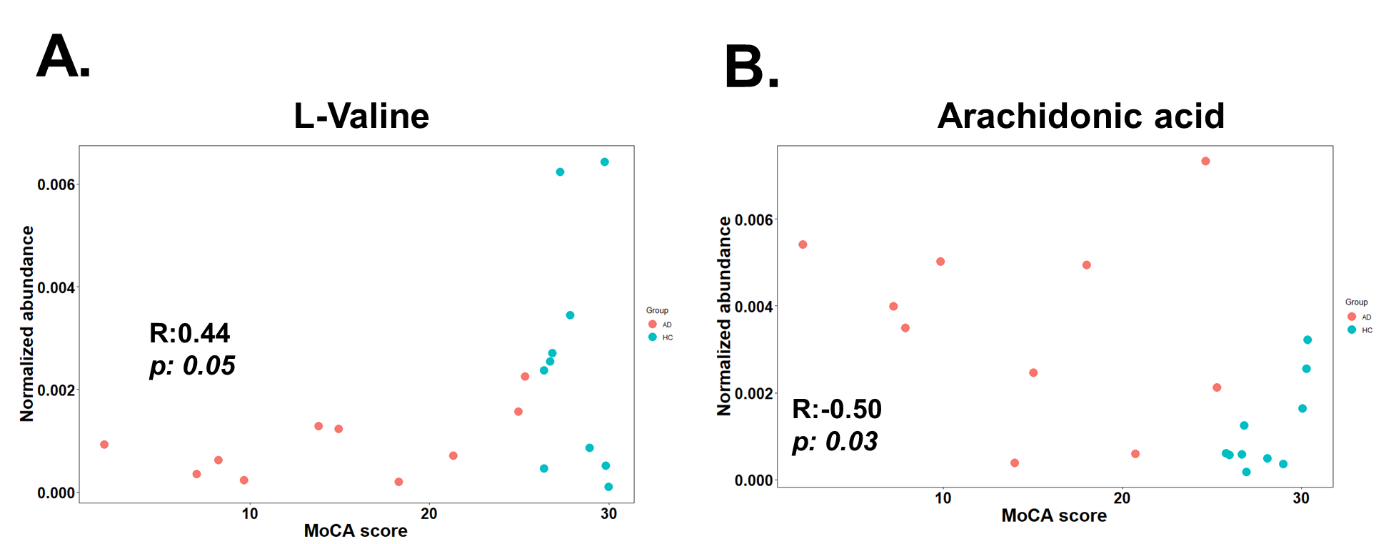


**Figure S2. The correlation analysis of normalized abundance and MoCA scores within 20 participants (10 patients with AD and 10 controls).** The Pearson correlation analysis between normalized abundance of metabolites and MoCA scores was conducted by R 4.3.0. The correlation coefficient values were 0.44 (p = 0.05) and -0.50 (p = 0.03) for **A.** L-valine and **B.** arachidonic acids, respectively. The statistically significant correlation coefficients indicate a noteworthy association, where higher levels of L-valine and lower levels of arachidonic acid may relate to the impairment of cognitive function. This finding indicated that the combination of these two metabolites might be used as an early indicator in AD diagnosis and prevention.

**Table S6. The detail clinical characteristics for each participant.**

| Patient number | Group | Sex | Age | MoCA score | BMI | Smoke | Alcohol intake | Family medical history | Cosmetic treatment |
| --- | --- | --- | --- | --- | --- | --- | --- | --- | --- |
| C0015 | AD | M | 75 | 7 | 33.3 | past | No | No | Never |
| C0066 | AD | F | 59 | 21 | 22.6 | No | No | No | Dyeing |
| C0072 | AD | F | 68 | 25 | 24.9 | No | No | No | Never |
| C0074 | AD | M | 80 | 8 | 22.4 | past | No | No | Never |
| C0075 | AD | F | 71 | 25 | 26.0 | No | No | No | Never |
| C0085 | AD | F | 77 | 10 | 17.7 | No | No | No | Never |
| C0098 | AD | F | 78 | 14 | 22.3 | No | No | Y (Mother and sister) | Perming and Dyeing |
| C0113 | AD | F | 68 | 2 | 22.0 | No | No | No | Dyeing |
| C0120 | AD | F | 83 | 15 | 27.2 | No | No | No | Perming |
| C0128 | AD | M | 86 | 18 | 22.2 | No | No | Y (Brother) | Perming |
| H0113 | HC | F | 62 | 30 | 25.2 | No | No | No | Perming and Dyeing |
| H0144 | HC | M | 72 | 27 | 24.7 | No | No | No | Never |
| H0154 | HC | F | 56 | 29 | 19.6 | No | No | No | Perming |
| H0156 | HC | F | 57 | 30 | 22.3 | No | No | Y (Mother) | Dyeing |
| H0162 | HC | M | 72 | 27 | 27.5 | past | past | No | Never |
| H0175 | HC | M | 70 | 26 | 27.5 | No | No | No | Never |
| H0182 | HC | M | 85 | 26 | 20.6 | No | No | No | Never |
| H0193 | HC | F | 75 | 28 | 20.3 | No | No | No | Dyeing |
| H0199 | HC | F | 70 | 27 | 22.1 | No | No | No | Perming and Dyeing |
| H0207 | HC | F | 70 | 30 | 25.0 | No | No | No | Never |

**Reference**

(1) Simard, M.; Archambault, A.-S.; Lavoie, J.-P. C.; Dumais, É.; Di Marzo, V.; Flamand, N. Biosynthesis and metabolism of endocannabinoids and their congeners from the monoacylglycerol and N-acyl-ethanolamine families. *Biochemical Pharmacology* **2022**, *205*, 115261.
